# Electroacupuncture for treating the cognitive symptoms of Alzheimer’s disease: a randomized controlled trial

**DOI:** 10.3389/fpsyt.2026.1834514

**Published:** 2026-05-04

**Authors:** Ran Li, Zehao Chen, Yuhang Jiang, Shanshan Yan, Jiakai He, Jinrong Yan, Guanhua Zong, Zongxi Yi, Xinyu Ren, Baohui Jia

**Affiliations:** 1 Department of Rehabilitation Medicine, Guang’anmen Hospital, China Academy of Chinese Medical Sciences, Beijing, China; 2 Guang’anmen Hospital, China Academy of Chinese Medical Sciences, Beijing, China; 3 Department of Traditional Chinese Medicine, Peking University People’s Hospital, Beijing, China; 4 Guang'anmen Hospital, Beijing University of Chinese Medicine, Beijing, China

**Keywords:** Alzheimer’s disease, cognitive symptoms, electroacupuncture, nonpharmacological interventions, randomized controlled trial

## Abstract

**Introduction:**

The long-term efficacy of electroacupuncture (EA) in treating cognitive symptoms of Alzheimer’s disease (AD) remains unclear, and its time-dependent relationship requires further investigation.

**Methods:**

Sixty-six patients were allocated to the EA or sham EA group, with stimulation for 20 minutes, for a 24-week treatment period, and were followed-up at 4 weeks. The primary outcome was the mean change in the Alzheimer’s Disease Assessment Scale–Cognitive (ADAS-Cog) score. Activities of daily living and behavioral and psychological symptoms were also assessed.

**Results:**

After 24 weeks of intervention, patients demonstrated significant improvements in ADAS-Cog scores, with sustained benefits observed during the 4-week follow-up period. In addition, improvements were observed in activities of daily living and behavioral and psychological symptoms.

**Discussion:**

The use of EA as a promising nonpharmacological intervention for AD.

**Clinical Trial Registration:**

https://www.chictr.org.cn/showproj.html?proj=151275, identifier (ChiCTR2200056329).

## 1 Introduction

With the acceleration of the aging population, dementia has emerged as a severe public health challenge, posing a significant threat to the physical and mental well-being of the elderly. In 2019, the global number of individuals living with dementia was estimated at 57.4 million, with projections indicating a rise to 152.8 million cases by 2050 (1). Alzheimer’s disease (AD), accounting for approximately two-thirds of all dementia cases, is a progressive neurodegenerative disorder that is clinically characterized by cognitive decline, impaired activities of daily living, and neuropsychiatric symptoms (2). As the most prevalent form of dementia, AD is expected to impose an escalating economic and social burden on patients, caregivers, and health care systems worldwide (3).

The currently available pharmacotherapies (e.g., donepezil and memantine) offer symptomatic relief but neither halt disease progression nor prevent longitudinal adverse effects, including nausea, vomiting, and dizziness (4, 5). These limitations have prompted increasing interest in nonpharmacological interventions that can be applied over longer durations. Acupuncture is a nonpharmacological intervention that has been shown to have beneficial effects on AD in multiple studies (6–8) and has been recognized as a potential therapeutic strategy by the Chinese Alzheimer’s Disease Prevention and Control Association (9). Among commonly selected acupoints, GV20, EX-HN1, EX-HN3, and GB20 are frequently used in clinical and experimental studies (10). Emerging evidence from preclinical studies suggests that acupuncture at GV20 may reduce β-amyloid (Aβ1–42) deposition in the hippocampus, inhibit neuronal apoptosis, and improve cognitive performance (11). Additional experimental studies indicate that stimulation at GV20 and EX-HN3 may modulate multiple pathological processes relevant to AD, including enhancement of synaptic plasticity, increased synaptic density, regulation of cholinergic neurotransmission, inhibition of tau hyperphosphorylation, and attenuation of neuroinflammatory responses (12). Neuroimaging studies further suggest that acupuncture may influence brain regions implicated in cognitive function, including the hippocampus, prefrontal cortex, cingulate gyrus, and temporal and parietal cortices—regions that are critically involved in memory and attention and are commonly affected in AD (13). Moreover, acupuncture at GV20 and EX-HN1 has been reported to enhance functional connectivity between the hippocampus and the default mode network, as well as sensory and visual processing regions (14). However, it should be noted that most of these mechanistic findings are derived from preclinical or exploratory studies, and their direct relevance to clinical populations remains to be established.

Compared with manual acupuncture (MA), electroacupuncture (EA) demonstrates superior efficacy in treating neurologic disorders (15). Meta-analytic evidence has further indicated that a standalone EA intervention produces significantly greater improvements in the Mini-Mental State Examination (MMSE) scores of patients with AD than MA alone (16).

Despite these promising findings, an evidence map has shown that only a small proportion of acupuncture trials in AD have incorporated sham controls, which may limit the ability to adequately account for placebo effects (17). Additionally, 87.16% of the studies had observation periods spanning 3 months or less, and thus the longitudinal efficacy of EA is difficult to ascertain (17). Preliminary evidence has also suggested that longer treatment duration may be associated with greater clinical improvement, highlighting the importance of examining time-dependent treatment effects (18).

To overcome these limitations, a randomized, sham-controlled trial with blinded participants, outcome assessors, and data analysts was conducted to compare 24 weeks of EA with sham EA in patients with AD. The primary aim of this trial was to assess longitudinal changes in cognitive performance while simultaneously examining the temporal pattern of response to EA treatment over a 24-week period.

## 2 Methods

### 2.1 Study design

This single-center, parallel-group, patient- and outcome-assessor-blinded randomized clinical trial was designed by the investigators. Eligible participants were randomly assigned 1:1 to receive the EA and sham EA interventions. This study consisted of a 24-week treatment phase followed by a 4-week follow-up and was conducted at Guang’anmen Hospital, China Academy of Chinese Medical Sciences (CACMS), from December 17, 2021, to December 31, 2024. The study protocol was approved by the Institutional Review Board at Guang’anmen Hospital, CACMS (No. 2021-124-KY-01), and was registered at the Chinese Clinical Trial Registry (No. ChiCTR2200056329). All participants and their legal representatives provided written informed consent. This trial was conducted in accordance with the Declaration of Helsinki (2013 revision) and is reported in accordance with the CONsolidated Standards Of Reporting Trials (CONSORT) guidelines.

### 2.2 Study population and recruitment

Eligible participants with AD were recruited from the outpatient clinic of the Department of Rehabilitation Medicine and Acupuncture at Guang’anmen Hospital, CACMS, as well as through posters, advertisements, and official hospital WeChat accounts. Eligibility screens and clinical evaluations were conducted by licensed physicians in the Departments of Encephalopathy and Neurology. The inclusion criteria were as follows: a diagnosis of probable AD based on the National Institute on Aging–Alzheimer’s Association criteria (19); age between 50 and 85 years; a Clinical Dementia Rating (CDR) score of 0.5 or higher; a MMSE score of 26 or lower; and a Hachinski Ischemic Scale (HIS) score of 4 or lower. The exclusion criteria included the following: other neurological or systemic disorders that may cause progressive cognitive impairment; recent use of medications or exposure to substances known to impair cognition; a history of trypanophobia or active skin infections; acupuncture or EA treatment within the past 2 weeks; and participation in other clinical trials. Participants in the study received appropriate transportation subsidies.

### 2.3 Randomization and blinding

Eligible participants were randomly assigned in a 1:1 ratio to either the EA group or the sham EA group using a computer-generated sequence with IBM SPSS Statistics (version 26.0, IBM Corp). Cards containing the randomization information were sealed in sequentially numbered opaque envelopes by an independent research assistant. A clinic nurse who was blinded to the trial procedures opened each envelope sequentially according to the enrollment order to allocate each participant to one of the two treatments.

Both groups received their interventions in a private treatment room in the supine position, with no accompanying personnel permitted during treatment. Identical EA devices were used in both groups. In the sham EA group, the lead terminals were internally disconnected prior to fixation, preventing electrical current delivery while maintaining identical visual appearance to the active devices. Detailed descriptions of the EA devices are provided in Supplement 2.

Participants, outcome assessors, and data analysts were blinded to group allocation throughout the study, whereas the acupuncturists were not blinded due to the nature of the intervention. Due to cognitive impairment, many participants had limited ability to reliably recall or distinguish details of the intervention they had received, which limited the feasibility of conducting formal blinding assessments. Therefore, no formal evaluation of blinding credibility was performed. This should be considered when interpreting the results, as incomplete verification of blinding may introduce potential performance or expectation bias.

### 2.4 Intervention and control

The trial protocol was developed on the basis of a previous pilot study (20). Disposable stainless steel filiform needles (0.3 mm × 25 mm and 0.3 mm × 40 mm; Huatuo brand, Suzhou Medical Appliance Factory) were used with JS-502-A EA devices (Wuxi Shenping Xintai Medical Technology Co., Ltd). Both groups received interventions in cephalic regions, with the sham EA administered at nonacupoint locations positioned 2 cm lateral to standard acupoint sites. All procedures were conducted according to a standardized protocol by licensed acupuncturists with at least 2 years of clinical experience, following uniform training prior to the trial. Details on the intervention protocols and electrode placement schematics are provided in Supplement 2. The bidirectional symmetrical pulse wave stimulation parameters for the protocol used for the EA group included dilatational waves at 2/100 (each wave for 3 seconds, alternately), pulse widths of 0.6 ms ± 30% at 2 Hz and 0.2 ms ± 30% at 100 Hz, and the stimulation intensity adjusted to the patients’ maximum tolerable level (0.1–5 mA).

To enhance the credibility of blinding while minimizing specific therapeutic effects, the sham electroacupuncture protocol was designed according to commonly used control strategies in acupuncture trials. Specifically, needles were inserted at nonacupoint locations approximately 2 cm lateral to the predefined acupoints, avoiding recognized meridians. In addition, identical electroacupuncture devices were used in both groups; in the sham group, the internal leads were disconnected to prevent current delivery while preserving identical visual appearance and operational procedures. However, this approach was selected to balance methodological rigor and participant blinding and is consistent with prior randomized controlled trials in acupuncture research.

All patients underwent the intervention for 20 minutes per session, three times a week on nonconsecutive days, for 24 weeks, followed by a 4-week follow-up period. Both groups continued standard pharmacologic treatment with donepezil and memantine, administered according to the National Institute for Health and Care Excellence (NICE) guidelines (21). Any changes in concomitant medications during the trial were documented. Participants were instructed not to initiate any new nonpharmacological interventions targeting cognitive function (e.g., cognitive training or other neuromodulation therapies) during the study period.

### 2.5 Outcome measurements

The primary outcome measure was the change in the Alzheimer’s Disease Assessment Scale–Cognitive (ADAS-Cog) score at baseline and at weeks 8, 16, 20, 24, and 28 (22). The ADAS-Cog is a 12-item scale that evaluates patients through four cognitive domains commonly affected in AD: memory, language expression, praxis, and attention. A higher score indicates more severe cognitive impairment. A between-group difference of 4 points in ADAS-Cog scores was identified previously as the minimal clinically important difference (MCID) in this measure (23). Secondary outcome measures included comparisons of the activities of daily living (ADL) scale score (24), and the Neuropsychiatric Inventory (NPI) score (25) between the EA and sham EA groups at baseline, week 24, and week 28. Adverse events related to the EA treatments during the trial were reported by patients and their caregivers and documented by the study physicians. Adherence to the intervention was monitored by recording attendance at each treatment session.

### 2.6 Sample size calculations

As noted above, a total between-group difference of 4 points on the ADAS-Cog at 24 weeks was defined as clinically meaningful according to previous studies and expert consensus (26). Assuming a standard deviation of 5, with a two-sided α of 0.05 and 80% power, the estimated sample size was 26 participants per group. The target enrollment was set at 66 participants to accommodate an expected dropout rate of 20%.

### 2.7 Statistical analysis

The primary outcome was analyzed using a linear mixed-effects model with a random intercept to account for subject-level heterogeneity. The model included fixed effects for treatment group, visit, and treatment-by-visit interaction, with an unstructured covariance matrix for repeated measures within each participant. The outcome variable was the change from baseline at each scheduled postbaseline visit (i.e., postbaseline value minus baseline value). Least-squares mean changes from baseline at each visit were estimated from the treatment-by-visit interaction, along with corresponding 95% confidence intervals and two-sided P values for treatment comparisons. In addition to the primary analysis based on the intention-to-treat population, sensitivity analyses were conducted using the per-protocol population to assess the robustness of the results. The per-protocol population included participants who completed the intervention with adequate adherence and without major protocol deviations.

The same modeling approach was applied to other continuous secondary outcomes, including scores on the ADL scale and the NPI. Adherence, blinding, and adverse event data were summarized descriptively. All analyses followed the intention-to-treat principle and were conducted using R version 4.4.3 (R Foundation for Statistical Computing). A two-sided *P* value of less than.05 was considered to indicate statistical significance. No adjustments were made for multiple comparisons; therefore, all analyses of secondary outcomes were considered exploratory and hypothesis-generating. The study was not powered to detect differences in secondary endpoints, and these results should be interpreted with caution.

## 3 Results

### 3.1 Participants

**Figure 1:**
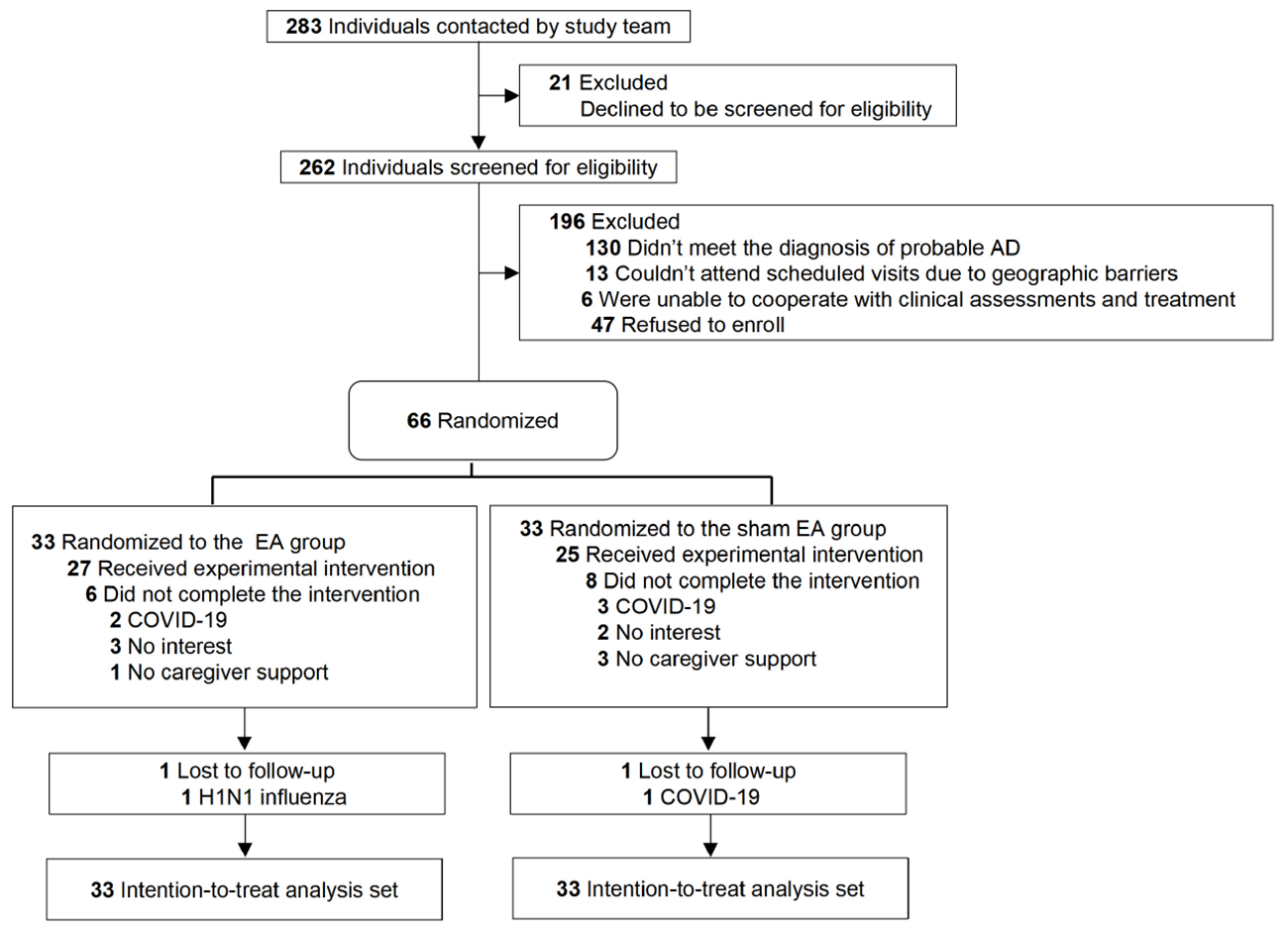
Study flowchart. AD, Alzheimer disease; EA, electroacupuncture; COVID-19, Coronavirus Disease 2019; H1NI influenza, Hemagglutinin 1 Neuraminidase 1 influenza.

We contacted 283 potential participants between December 2021 and December 2024, among whom 21 declined to be screened; the remaining 262 subsequently underwent eligibility screening, of whom 130 did not meet the diagnostic criteria for probable AD, 13 lived too far from the hospital, 3 had a severe hearing impairment precluding the completion of the scale assessments, 3 were unable to cooperate with the intervention, and 47 declined participation. A total of 66 patients were randomly assigned to one of two groups: 33 in the EA group (mean [SD] age, 70.58 [8.53] years; 15 [45.5%] female) and 33 in the sham EA group (mean [SD] age, 72.97 [7.24] years; 19 [57.6%] female). The completion rates for the 24-week treatments and 4-week follow-up assessments were 78.8% (26 of 33) in the EA group and 72.7% (24 of 33) in the sham EA group. There were no differences in any of the baseline variables between the groups. The participant recruitment flowchart is shown in **Figure 1** (see CONSORT diagram), and the baseline demographics and characteristics of the patients are summarized in **Table 1**.

**Table 1:** Baseline demographics and characteristics of the patients.

Characteristics	EA Group (n=33)	Sham EA Group (n=33)
Demographics		
Mean age (SD), y	70.58 ± 8.53	72.97 ± 7.24
Sex, *n* (%)		
Male	18 (54.5%)	14 (42.4%)
Female	15 (45.5%)	19 (57.6%)
Education level, *n* (%)		
Primary school	2 (6.1%)	1 (3.0%)
Junior high school	9 (27.3%)	10 (30.3%)
Senior high school	10 (30.3%)	8 (24.2%)
College or higher	12 (36.4%)	14 (42.4%)
Occupation, *n* (%)		
Manual labor	12 (36.4%)	9 (27.3%)
Intellectual labor	21 (63.6%)	24 (72.7%)
Clinical Features		
Mean disease duration (SD), M	47.06 ± 28.28	52.89 ± 23.63
Mean number of comorbidities (SD)	1.36 ± 0.96	1.12 ± 1.32
Mean number of AD medications (SD)	0.85 ± 0.36	0.88 ± 0.33
MMSE score (mean ± SD)	17.18 ± 7.13	14.82 ± 6.86
CDR score, *n* (%)		
0.5	6 (18.2%)	2 (6.1%)
1	12 (36.4%)	9 (27.3%)
2	12 (36.4%)	16 (48.5%)
3	3 (9.1%)	6 (18.2%)
Hachinski score, mean (SD)	1.39 ± 1.20	1.27 ± 1.21
PHQ-9 score, mean (SD)	1.12 ± 1.43	1.61 ± 2.75
ADAS-Cog score, mean (SD)	26.42 ± 17.49	31.15 ± 18.42
ADL score, mean (SD)	37.18 ± 14.16	39.36 ± 13.62
BADL score, mean (SD)	11.79 ± 4.44	12.15 ± 4.94
IADL score, mean (SD)	25.39 ± 10.22	27.21 ± 9.22
NPI-Frequency × Severity score, mean (SD)	16.52 ± 18.18	13.64 ± 12.95
NPI-Frequency score, mean (SD)	7.58 ± 7.78	7.03 ± 6.12
NPI-Severity score, mean (SD)	6.30 ± 6.24	5.45 ± 4.52
NPI-Caregiver Distress score, mean (SD)	5.58 ± 5.59	6.09 ± 5.48

EA, electroacupuncture; ADAS-Cog, Alzheimer’s Disease Assessment Scale–Cognitive; ADL, Activities of Daily Living; BADL, basic ADL; IADL, instrumental ADL; MMSE, Mini-Mental State Examination; CDR, Clinical Dementia Rating; PHQ-9, Patient Health Questionnaire-9; NPI, Neuropsychiatric Inventory.

### 3.2 Primary efficacy endpoint

**Figure 2:**
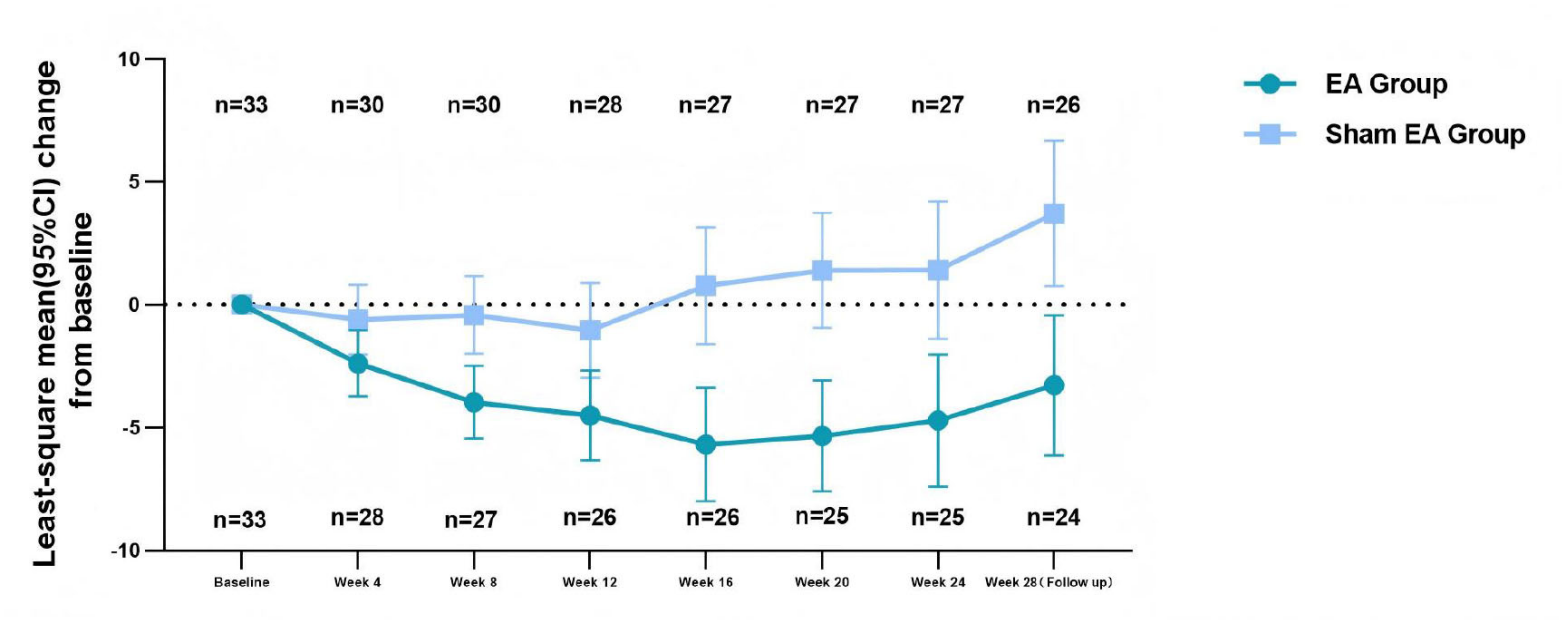
Least-square mean change of ADAS-Cog score from baseline to week 28. CI, Confidence Interval; ADAS-Cog, Alzheimer’s Disease Assessment Scale–Cognitive; EA, electroacupuncture.

For the primary endpoint, the least-square mean change in the ADAS-Cog scale score from baseline to week 24 was -4.71 (95% CI, -7.40 to -2.02) in the EA group versus 1.40 (95% CI, -1.40 to 4.21) in the sham EA group. At week 24, compared with the sham group, the EA group exhibited a significantly greater, clinically meaningful reduction in ADAS-Cog scale score (mean difference, -6.12 [95% CI, -10.00 to -2.23]; *P* <.01; MCID = 4) (**Table 2**). Compared with the sham EA group, the EA group exhibited a statistically significant improvement in the ADAS-Cog score starting at week 8 (mean difference, -3.55 [95% CI [-5.70 to -1.40]; *P* <.005), which decreased slightly but remained statistically significant at week 12. (mean difference, -3.45, 95% CI [-6.12 to -0.79]; *P* <.05), increased further at week 16 (mean difference, -6.46, 95% CI [-9.78 to -3.14]; *P* <.001), and week 20 (mean difference, -6.73, 95% CI [-9.99, -3.48]; *P* <.001). The effect was sustained during follow-up at week 28 (mean difference, -6.99; 95% CI [-11.09, -2.89]; *P* <.05) (**Figure 2**).

**Table 2:** Comparison of primary and secondary outcomes between the two groups.

Outcomes	Sham EA group (n=33)	EA group (n=33)	Difference (95% CI)	*P* value
Least-square mean changes in the ADAS-Cog score from the baseline (95% CI)				
week 4	-0.61 (-2.02, 0.81)	-2.39 (-3.73, -1.04)	-1.78 (-3.74, 0.18)	0.0736
week 8	-0.42 (-1.99, 1.15)	-3.97 (-5.44, -2.49)	-3.55 (-5.70, -1.40)	0.0017
week 12	-1.05 (-2.98, 0.88)	-4.51 (-6.35, -2.67)	-3.45 (-6.12, -0.79)	0.0121
week 16	0.77 (-1.61, 3.16)	-5.69 (-7.99, -3.38)	-6.46 (-9.78, -3.14)	0.0003
week 20	1.39 (-0.95, 3.74)	-5.34 (-7.60, -3.08)	-6.73 (-9.99, -3.48)	0.0001
week 24	1.40 (-1.40, 4.21)	-4.71 (-7.40, -2.02)	-6.12 (-10.00, -2.23)	0.0026
week 28	3.71 (0.76, 6.67)	-3.28 (-6.12, -0.44)	-6.99 (-11.09, -2.89)	0.0012
Least-square mean changes in ADL total and subscale scores from the baseline (95% CI)				
ADL scale				
week 24	-6.49 (-13.42, 0.44)	-10.02 (-16.95, -3.09)	-3.53 (-13.35, 6.29)	0.4749
week 28	-8.40 (-15.55, -1.26)	-8.96 (-16.11, -1.82)	-0.56 (-10.68, 9.56)	0.9121
BADL scale				
week 24	1.33 (0.20, 2.45)	-0.38 (-1.46, 0.71)	-1.70 (-3.26, -0.14)	0.0331
week 28	1.57 (0.46, 2.68)	0.31 (-0.75, 1.38)	-1.26 (-2.79, 0.28)	0.1064
IADL scale				
week 24	3.13 (0.78, 5.48)	-1.34 (-3.60, 0.92)	-4.46 (-7.73, -1.20)	0.0084
week 28	2.69 (0.64, 4.74)	0.27 (-1.70, 2.24)	-2.42 (-5.27, 0.43)	0.0941
Least-square mean changes in NPI total and subscale scores from the baseline (95% CI)				
NPI-Frequency×Severity				
week 24	0.29 (-4.50, 5.09)	-6.65 (-11.26, -2.04)	-6.94 (-13.63, -0.26)	0.0421
week 28	0.59 (-3.96, 5.15)	-5.45 (-9.83, -1.07)	-6.04 (-12.39, 0.31)	0.0616
NPI-Frequency				
week 24	0.26 (-1.72, 2.25)	-2.98 (-4.89, -1.07)	-3.25 (-6.00, -0.49)	0.0220
week 28	0.36 (-1.62, 2.34)	-2.11 (-4.01, -0.20)	-2.46 (-5.21, 0.28)	0.0777
NPI- Severity				
week 24	0.27 (-1.23,1.77)	-2.54 (-3.98, -1.09)	-2.81 (-4.90, -0.71)	0.0096
week 28	-0.21 (-1.70,1.28)	-1.75 (-3.18, -0.32)	-1.54 (-3.61, 0.53)	0.1419
NPI-Caregiver Distress				
week 24	-0.26 (-1.77, 1.25)	-2.42 (-3.88, -0.96)	-2.16 (-4.26, -0.06)	0.0443
week 28	-0.20 (-1.73, 1.34)	-1.82 (-3.30, -0.34)	-1.62 (-3.76, 0.51)	0.1325

EA, electroacupuncture; ADAS-Cog, Alzheimer’s Disease Assessment Scale–Cognitive; ADL, Activities of Daily Living; BADL, basic ADL; IADL, instrumental ADL; NPI, Neuropsychiatric Inventory.

### 3.3 Secondary efficacy endpoint

In the ADAS-Cog word recall task, the EA group demonstrated significant improvements over the sham group at weeks 16 and 20. With respect to word recognition, the EA group showed sustained improvements from week 4 through week 28 (that is, at follow-up). Significant benefits in attention task performance were observed in patients treated with EA at week 8 and from week 16 to week 28 (Supplement 1).

**Figure 3:**
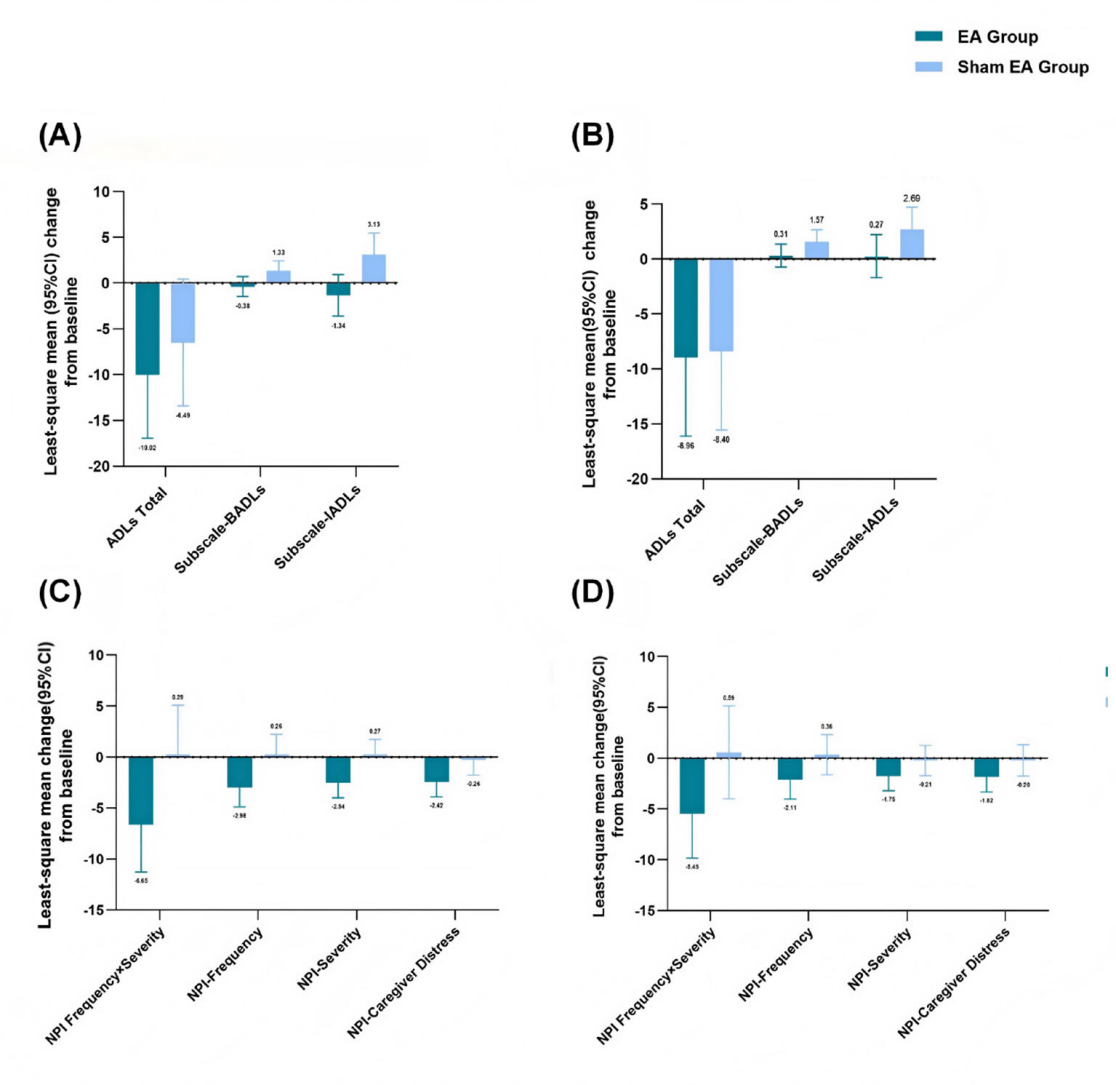
Least-square mean change of ADLs and NPI score **(A)** Least-square changes in ADLs from baseline to week 24. **(B)** Least-square changes in ADLs from baseline to week 28. **(C)** Least-square changes in NPI from baseline to week 24. **(D)** Least-square changes in NPI from baseline to week 28. CI, Confidence Interval; BADLs, Basic ADLs; IADLs, Instrumental ADLs; NPI, Neuropsychiatric Inventory; EA, electroacupuncture.

No significant between-group differences were observed in total ADL scores at week 24 or 28 (**Figure 3**). However, the least-square mean change in the basic (BADL) and instrumental ADL (IADL) scores of the EA group from the baseline was significantly greater than that in the sham EA group, with between-group differences of -1.70 (95% CI, -3.26 to -0.14; *P* <.05) and -4.46 (95% CI, -7.73 to -1.20; *P* <.05), respectively, at week 24. This improvement was not maintained at week 28, however (**Figure 3**).

Similar patterns were observed for the NPI outcomes. Compared with the sham EA group, the EA group showed significantly greater improvements at week 24, with mean between-group differences of -6.94 (95% CI, -13.63 to -0.26; *P* <.05) in the total NPI score; -3.25 (95% CI, -6.00 to -0.49; *P* <.05) in the NPI-Frequency subscale score; -2.81 (95% CI, -4.90 to -0.71; *P* <.05) in the NPI-Severity subscale score; and -2.16 (95% CI, -4.26 to -0.06; *P* <.05) in the NPI-Caregiver Distress subscale score. However, no significant between-group differences were observed in the total NPI scores at week 28 (**Figure 3**).

### 3.4 Sensitivity analysis

Sensitivity analyses using the per-protocol (PP) population yielded results that were consistentwith those of the intention-to-treat (ITT) analysis (see **Supplementary Table 1**). Specifically, in the PP analysis, the EA group showed significantly greater improvements in ADAS-Cog scores compared with the sham EA group from week 8 onward, with a between-group difference of −6.16 points (95% CI, −9.14 to −3.17; P = 0.0002) at week 24, which was comparable to the ITT estimate (−6.12 points; 95% CI, −10.00 to −2.23; P = 0.0026).

The temporal pattern of treatment effects observed in the ITT analysis—emerging at week 8 and increasing over time—was similarly observed in the PP analysis.

Overall, the consistency between ITT and PP analyses supports the robustness of the primary outcome findings.

### 3.5 Safety

Adverse events occurred in 12 patients in the EA group and 7 patients in the sham EA group. No adverse events attributed to the intervention were reported in the two groups during the study period (**Table 3**).

**Table 3:** Adverse events related and unrelated to treatment.

Adverse Event	EA group (n=33)		Sham EA group (n=33)
Unrelated to treatment			
Ocular discomfort	1 (3.03%)	0	
Knee injury	1 (3.03%)	0	
Fall	2 (6.06%)	2 (6.06%)	
Fall on a moving bus	1	0	
Nocturnal toileting-associated fall	0	2	
Fall down stairs	1	0	
Gait instability	1 (3.03%)	0	
Epigastric discomfort	0	1 (3.03%)	
Fatigue	1 (3.03%)	1 (3.03%)	
Diarrhea	1 (3.03%)	0	
COVID-19	2 (6.06%)	2 (6.06%)	
Common cold	2 (6.06%)	1 (3.03%)	
Mild overheating	1	0	
Total	12 (36.36%)	7 (21.21%)	
Serious Adverse Events	0	0	

EA, electroacupuncture.

### 3.6 Adherence to intervention

The planned intervention consisted of 72 sessions over 24 weeks. The mean number of completed sessions was 54.21 ± 19.78 in the EA group and 50.03 ± 21.88 in the sham EA group, with no significant difference between groups (P = 0.230).

Treatment interruptions were recorded and included factors such as holidays, weather conditions, COVID-19–related disruptions, and other objective reasons. There were no statistically significant between-group differences in the number of sessions affected by these factors (all P > 0.05), suggesting comparable adherence between groups (**Table 4**).

**Table 4:** Treatment adherence and influencing factors in the two groups.

Intervention	Total number of treatment sessions (Mean ± SD)	Sessions affected by holidays (Mean ± SD)	Sessions affected by weather (Mean ± SD)	Sessions affected by pandemic (Mean ± SD)	Total sessions affected by objective factors (Mean ± SD)
EA Group	54.21 ± 19.78	1.89 ± 1.32	0.30 ± 0.64	1.33 ± 2.38	3.52 ± 3.01
Sham EA Group	50.03 ± 21.88	2.36 ± 1.92	0.36 ± 0.74	1.91 ± 2.92	4.64 ± 3.97
Z	-1.201	-0.819	-0.289	-0.567	-0.809
p	0.230	0.413	0.773	0.571	0.418

## 4 Discussion

This randomized clinical trial revealed that a 24-week course of EA effectively improved cognitive function in patients with AD, with sustained benefits lasting for 4 weeks after treatment completion. Compared with the sham group, the EA group showed a statistically significant and clinically meaningful 6.12-point greater reduction in the ADAS-Cog score at week 24 relative to baseline, which exceeded the 4-point MCID threshold for the ADAS-Cog score observed for patients with AD in randomized controlled trials. A meta-analysis revealed greater improvements in ADAS-Cog scores when the treatment duration was extended from ≤8 to 9–12 weeks (2.08 vs. 4.37 points), suggesting that longer treatment duration may be associated with greater clinical improvement (27). These findings provide insight into the temporal dynamics of response to EA and preliminary evidence supporting the potential cognitive benefits of EA for AD.

The between-group differences in ADAS-Cog scores followed a temporal pattern, with effects emerging at week 8, reaching clinically meaningful levels by week 16, and peaking at week 20. This pattern may reflect a time-dependent response under the fixed EA treatment protocol (18), a finding consistent with that of a previous meta-analysis (28). However, given the use of a single fixed treatment schedule, it is not possible to distinguish treatment-related temporal effects from other time-dependent factors, such as the natural progression of AD, or repeated testing effects. In addition, without neuroimaging or biomarker data, the observed improvements cannot be directly interpreted as evidence of underlying neuronal changes and may partly reflect nonspecific factors such as test familiarity or general arousal. Furthermore, this study showed that the therapeutic effects of EA could be sustained for at least 4 weeks after treatment completion.

Beyond global cognition, exploratory analyses suggested improvements in specific cognitive domains, particularly episodic memory, as reflected by enhanced performance in word recall and word recognition tasks. Word recall requires unprompted retrieval of learned words, which requires the engagement of free recall processes that involve prefrontal cortical and hippocampal information encoding, storage, and retrieval (29–32), whereas word recognition tests familiarity and recollection processes, which are linked to the entorhinal cortex, the surrounding limbic cortex, hippocampus and medial temporal lobe (33–35). Improvements in attention were also observed. While these findings may suggest a potential influence on neural circuits involved in memory and attention, such interpretations remain speculative in the absence of mechanistic assessments.

With respect to secondary outcomes, no significant between-group difference was observed in overall ADL scores at week 24, which contrasts with some prior studies reporting functional improvements following acupuncture (36). This discrepancy may be attributable to the limited sample size and dilution of the placebo effect caused by the “basic medication combined with sham EA” control design. Subscale analyses indicated improvements in instrumental activities of daily living (IADL), which rely on higher-order cognitive functions and are often affected earlier in the disease course (37, 38). These findings suggest a potential role of EA in preserving complex functional abilities, although they should be interpreted as exploratory. Nonpharmacological therapies are recommended as the initial approach for managing the behavioral and psychological symptoms (BPSs) of AD patients (39). In this study, EA treatment was associated with reductions in neuropsychiatric symptoms, as reflected by improvements in NPI scores, which are consistent with those of a previous study on the efficacy of acupuncture in treating BPS (40). Given the multifactorial neurobiological basis of these symptoms, including neurotransmitter dysregulation and network-level dysfunction (41) (42, 43), these results warrant further investigation in larger studies (44).

It is also important to consider these findings within the context of existing literature. Previous studies on acupuncture for dementia have yielded heterogeneous and sometimes inconsistent results, likely due to variations in study design, control conditions, treatment duration, and outcome measures. Therefore, the present findings should be interpreted cautiously and require confirmation in larger, multicenter trials to establish their generalizability and reproducibility.

This study has several limitations. First, as a single-center trial with a relatively small sample size and a notable dropout rate, the findings may be subject to selection and attrition bias and may have limited generalizability. Although a linear mixed-effects model was used under the intention-to-treat principle, which assumes that data are missing at random, this assumption may not fully hold. Reasons for discontinuation, including COVID-19–related disruptions and caregiver-related factors, may not be random and could affect the robustness of the results. Second, the absence of neuroimaging or biomarker assessments limits the ability to determine whether the observed improvements reflect underlying neuronal changes or are attributable to nonspecific factors such as repeated testing effects or general arousal. This may introduce diagnostic uncertainty and limit mechanistic interpretation. Third, although a sham-controlled design was employed, the sham intervention may not have been completely inert, and subtle somatosensory or expectancy effects cannot be excluded, and the observed between-group differences may represent conservative estimates of the true treatment effect. Fourth, the study was powered only for the primary outcome, and secondary analyses were not adjusted for multiple comparisons, and thus should be considered exploratory. Finally, although efforts were made to maintain blinding, the use of individualized stimulation intensity and the absence of formal blinding assessment raise the possibility of partial unblinding, which may introduce performance or expectation bias. Notably, any such bias would be expected to favor the active intervention; however, the consistency of effects across multiple time points provides some support for the robustness of the findings. The consistency between intention-to-treat and per-protocol analyses further supports the robustness of the primary findings, although these results should still be interpreted with caution given the sample size.

In summary, this study demonstrated that a 24-week EA treatment significantly improved cognitive function in patients with AD over sham EA. Analyses at multiple time points revealed that cognitive benefits emerged by week 8, reached clinically meaningful levels by week 16, and peaked at week 20, with effects persisting for at least 4 weeks after treatment. EA also showed potential improvements in instrumental activities of daily living and behavioral and psychological symptoms; however, these findings are exploratory and should be interpreted with caution. Overall, these results provide preliminary evidence supporting the potential role of EA as a nonpharmacological intervention for AD, while highlighting the need for larger, rigorously designed multicenter trials incorporating objective biomarkers to confirm these findings.

## Data Availability

The datasets generated and/or analyzed during the current study are not publicly available as research ethics permission is for the use of the data by the research team only. Data may, however, be available from the corresponding author on reasonable request. Requests to access the datasets should be directed to BJ, myrroossee@aliyun.com.

## Supplementary material

Supplementary Table 1 Comparison of Primary and Secondary Outcomes Between the 2 Groups (per-protocol PP analysis) Abbreviations: Alzheimer’s Disease Assessment Scale–Cognitive, ADAS-Cog; ADLs, Activities of Daily Living scale; BADL, Basic ADLs; IADL, Instrumental ADLs; NPI, Neuropsychiatric Inventory.
