# Supplementary material for: Electroacupuncture for treating the cognitive symptoms of Alzheimer’s disease: a randomized controlled trial
Source: Front Psychiatry. 2026 May 4;17:1834514. doi: 10.3389/fpsyt.2026.1834514 (PMC13180848; doi:10.3389/fpsyt.2026.1834514)
Supplement: Supplementary Table 1 — Comparison of Primary and Secondary Outcomes Between the 2 Groups (per-protocol PP analysis) Abbreviations: Alzheimer’s Disease Assessment Scale–Cognitive, ADAS-Cog; ADLs, Activities of Daily Living scale; BADL, Basic ADLs; IADL, Instrumental ADLs; NPI, Neuropsychiatric Inventory. [file SupplementaryFile1.docx]

**eFigure 1.** EA device and electrode placement schematics

**eTable 1.** Anatomical Localization of Acupoints and acupuncture manipulation

**eTable 2.** Comparison of ADAS-Cog Subscale Scores Between the 2 Groups

**eFigure 1. EA device and electrode placement schematics**


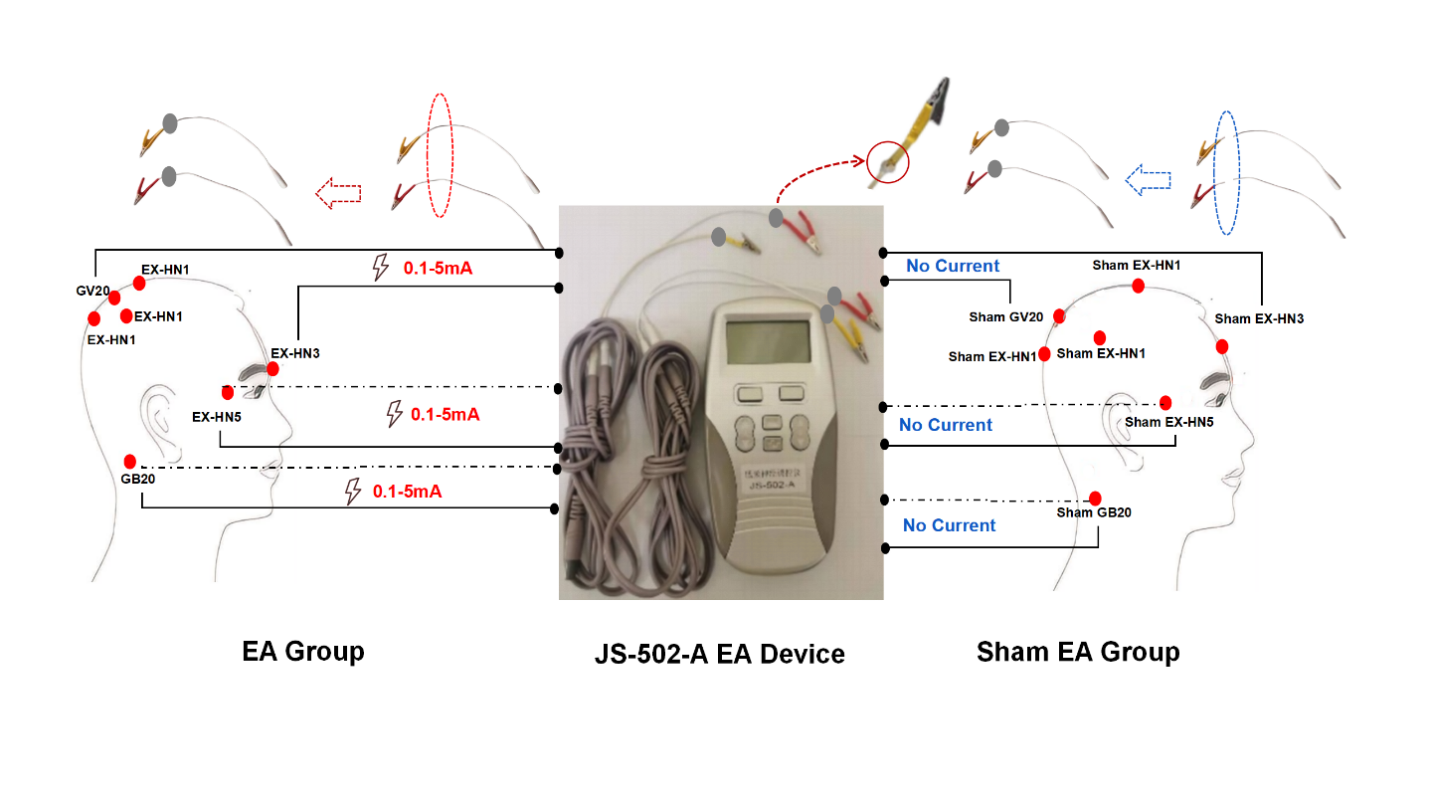


**Abbreviations:** EA, electroacupuncture.

**eTable 1. Anatomical Localization of Acupoints and acupuncture manipulation**

| **Acupoint** | **Location** | **Filiform Needle** | **Insertion** | **Manipulation** |
| --- | --- | --- | --- | --- |
| GV20 | On the head, at the midpoint of the line connecting the apexes of both ears. | 0.30 mm × 25 mm | 15-degree anterior subcutaneous insertion to 5 mm depth | Twirling technique until“*deqi”* sensation(a composite sensory response characterized by localized soreness, distention, or numbness) |
| EX-HN1 | Four points on the head, each 1 cun (≈1 inch) anterior, posterior, and lateral to GV20. | 0.30 mm × 25 mm | 15-degree anterior subcutaneous insertion to 5 mm depth | Twirling technique until“*deqi”* sensation |
| EX-HN3 | On the head, in the depression midway between the medial ends of the two eyebrows. | 0.30 mm × 25 mm | 15-degree insertion toward nasal tip to 5 mm depth | None |
| EX-HN5 | On the head, in the temporal depression approximately 1.5 cm posterior to the midpoint between the lateral end of the eyebrow and the outer canthus. | 0.30 mm × 40 mm | Perpendicular insertion to 10 mm depth | None |
| GB20 | In the posterior cervical region, inferior to the occipital bone, in the depression between the origins of the sternocleidomastoid and trapezius muscles. | 0.30 mm × 40 mm | insertion toward nasal tip to 20 mm depth | Twirling technique until“*deqi”* sensation |

**eTable 2. Comparison of ADAS-Cog Subscale Scores Between the 2**

**Group**

| **ADAS-Cog Subscale** | **EA Group (n=33)** | **Sham EA Group (n=33)** | **Difference (95%CI)** | ***P* value** |
| --- | --- | --- | --- | --- |
| **Least-square mean changes from baseline in word recall task (95% CI)** | | | | |
| week 4 | -0.88 (-3.03, 1.28) | -2.73 (-4.80, -0.66) | -1.86 (-4.84, 1.13) | 0.2286 |
| week 8 | -0.71 (-2.86, 1.44) | -4.46 (-6.53, -2.40) | -3.75 (-6.74, -0.77) | 0.0173 |
| week 12 | -1.04 (-3.19, 1.11) | -4.81 (-6.87, -2.74) | -3.77 (-6.75, -0.78) | 0.0169 |
| week 16 | 0.46 (-1.69, 2.61) | -5.96 (-8.03, -3.90) | -6.42 (-9.40, -3.44) | 0.0001 |
| week 20 | 1.21 (-0.94, 3.36) | -5.81 (-7.87, -3.74) | -7.02 (-10.00, -4.03) | 0.0000 |
| week 24 | 1.04 (-1.11, 3.19) | -5.12 (-7.18, -3.05) | -6.16 (-9.14, -3.17) | 0.0002 |
| week 28 | 3.42 (1.27, 5.57) | -3.73 (-5.80, -1.66) | -7.15 (-10.13, -4.17) | 0.0000 |
| **Least-square mean changes from baseline in** **word recognition (95% CI)** | | | | |
| week 4 | 0.18(-0.73, 1.09) | -1.13(-1.99, -0.26) | -1.31(-2.56, -0.05) | 0.0415 |
| week 8 | 0.11(-0.77, 0.99) | -1.32(-2.15, -0.50) | -1.43(-2.64, -0.22) | 0.0210 |
| week 12 | -0.22(-1.34, 0.91) | -2.11(-3.18, -1.04) | -1.89(-3.45, -0.34) | 0.0176 |
| week 16 | 0.58(-0.62, 1.78) | -2.38(-3.54, -1.22) | -2.97(-4.64, -1.29) | 0.0008 |
| week 20 | 1.02(-0.13, 2.17) | -2.16(-3.26, -1.06) | -3.18(-4.78, -1.59) | 0.0002 |
| week 24 | 0.88(-0.34, 2.10) | -2.26(-3.43, -1.10) | -3.15(-4.83, -1.46) | 0.0004 |
| week 28 | 1.47(0.22, 2.72) | -2.07(-3.26, -0.88) | -3.54(-5.27, -1.82) | 0.0001 |
| **Least-square mean changes from baseline in attention task (95% CI)** | | | | |
| week 4 | 0.25(-0.15, 0.65) | 0.06(-0.31, 0.44) | -0.19(-0.73, 0.36) | 0.5017 |
| week 8 | 0.40(0.07, 0.73) | -0.29(-0.60, 0.02) | -0.69(-1.14, -0.24) | 0.0034 |
| week 12 | 0.43(0.03, 0.83) | -0.08(-0.46, 0.31) | -0.51(-1.06, 0.04) | 0.0708 |
| week 16 | 0.55(0.26, 0.83) | -0.15(-0.42, 0.12) | -0.70(-1.09, -0.30) | 0.0008 |
| week 20 | 0.55(0.19, 0.92) | -0.33(-0.67, 0.02) | -0.88(-1.38, -0.37) | 0.0010 |
| week 24 | 0.86(0.47, 1.26) | -0.39(-0.77, -0.02) | -1.26(-1.80, -0.71) | <.0001 |
| week 28 | 0.75(0.36, 1.14) | -0.37(-0.74, 0.00) | -1.12(-1.66, -0.58) | 0.0001 |

**Abbreviations:** Alzheimer’s Disease Assessment Scale–Cognitive, ADAS-Cog.
